# Comparison of urine and blood NGAL for early prediction of delayed graft function in adult kidney transplant recipients: a meta-analysis of observational studies

**DOI:** 10.1186/s12882-019-1491-y

**Published:** 2019-08-02

**Authors:** Ya Mei Li, Yi Li, Lin Yan, Han Wang, Xiao Juan Wu, Jiang Tao Tang, Lan Lan Wang, Yun Ying Shi

**Affiliations:** 1 Department of Laboratory Medicine/Research Centre of Clinical Laboratory Medicine, West China Hospital, Sichuan University, Chengdu, China; 2 West China School of Medicine, West China Hospital, Sichuan University, Chengdu, China; 3 Department of Nephrology, West China Hospital, Sichuan University, Chengdu, China

**Keywords:** Kidney transplantation, Delayed graft function, NGAL, Predictive biomarker

## Abstract

**Background:**

Neutrophil gelatinase-assoicated lipocalin (NGAL) appears to be a promising proximal tubular injury biomarker for early prediction of delayed graft function (DGF) in kidney transplant recipients. However, its predictive values in urine and blood were varied among different studies. Here, we performed the meta-analysis to compare the predictive values of urine NGAL (uNGAL) and blood NGAL (bNGAL) for DGF in adult kidney transplant recipients.

**Methods:**

We systematically searched Medline, Cochrane library and Embase for relevant studies from inception to May 2018. The summary receiver operating characteristic (SROC) curves, the pooled sensitivity, specificity and diagnostic odds ratio (DOR) were used to evaluate the prognostic performance of uNGAL and bNGAL for the identification of DGF.

**Results:**

A total of 1036 patients from 14 eligible studies were included in the analysis. 8 studies focused on NGAL in urine and 6 reported NGAL in serum or plasma. The composite area under the ROC (AUC) for 24 h uNGAL was 0.91 (95% CI, 0.89–0.94) and the overall DOR for 24 h uNGAL was 24.17(95% CI, 9.94–58.75) with a sensitivity of 0.88 (95% CI, 0.75–0.94) and a specificity of 0.81 (95% CI, 0.68–0.89). The composite AUC for 24 h bNGAL was 0.95 (95% CI, 0.93–0.97) and the overall DOR for 24 h bNGAL was 43.11 (95% CI, 16.43–113.12) with a sensitivity of 0.91 (95% CI, 0.81–0.96) and a specificity of 0.86 (95% CI, 0.78–0.92).

**Conclusions:**

Urine and serum/plasma NGAL were valuable biomarkers for early identification of DGF in kidney transplantation. In addition, the bNGAL was superior to uNGAL in early prediction of DGF.

**Electronic supplementary material:**

The online version of this article (10.1186/s12882-019-1491-y) contains supplementary material, which is available to authorized users.

## Background

Delayed graft function (DGF), traditionally defined as the need for dialysis within 7 days after kidney transplantation, remains one of the major obstacles for patients to recover from transplantation during the postoperative course. It significantly prolongs hospitalization stay, increases medical costs, and even worsens patients with increased risk of chronic kidney diseases or graft loss in the first year after transplantation [1]. In addition, the incidence of DGF is highly ranged from 5 to 50% in deceased-donor recipients and from 4 to 10% in living-donor recipients [2]. Therefore early identification of DGF is warranted as it not only provides adequate time for clinicians to adjust therapeutic intervention to limit the further development of allograft injury, but also greatly reduces the economic burden of patients [3].

Previously, the requirement for dialysis, the failure of serum creatinine (Scr) to decrease, the graft histopathology and the urine output following transplantation have been applied to identify DGF in kidney transplant recipients during a few days after transplantation [4]. However, these criteria not only induce marked variation in the diagnosis of DGF, but also delay the identification of DGF, thus disturbing the timely medication adjustment in kidney transplant recipients. Therefore, a lot of efforts have been made to find novel ideal biomarkers that can detect DGF soon after transplantation. Among a variety of these potential biomarkers [5], neutrophil gelatinase associated lipocalin (NGAL), a kidney proximal tubular injury biomarker, has been intensively studied in urine and blood (serum/plasma) and appears to be a non-invasive and valuable marker of DGF with high sensitivity and specificity in many centers. However, whether urine (uNGAL) or blood NGAL (bNGAL) is superior to its blood or urine counterpart in predicting the occurrence of DGF remains unclear so far. Therefore, we conducted the present meta-analysis of 14 observational studies to compare the accumulative predictive values of uNGAL and bNGAL for early identification of DGF in kidney transplant recipients.

## Methods

### Data sources and literature search strategies

Two investigators (Y.M.L. and Y.L.) independently searched the literature in Medline (via Pubmed), Cochrane Library and Embase (via Ovid) electronic databases from inception to May 2018. Following medical subject headings or keywords were systematically searched without language restriction: “NGAL”, “neutrophil gelatinase-associated lipocalin”, “Lipocalin 2”, “delayed graft function”, “DGF” and “kidney transplantation”. In addition, reference lists of included articles were hand-searched to identify other relevant studies. All potentially relevant records were imported into Endnote X7.7 (Thomson Corporation, Connecticut, USA) for further management.

### Study selection and included criteria

Two reviewers (Y.M.L. and Y.L.) independently screened the titles and abstracts and further retrieved the full text of each potentially relevant study to determine study eligibility. Disagreements were resolved by consensus adjudication. Prospective and retrospective cohort studies were eligible for inclusion if they evaluated the predictive performance of urine, serum or plasma NGAL for DGF in adult kidney transplant recipients. Conference abstracts, reviews, editorials, commentaries, letters and studies without mandatory predictive variables including the area under the receiver operating characteristic curve (AUC), sensitivity and specificity were excluded.

### Data extraction and quality assessment

Two investigators (Y.M.L. and Y.L.) independently extracted the data from eligible articles according to previously prepared data sheet. The following information were included in the sheet: (1) Study and patient characteristics, including first author, publish year, study design, research country, sample resource and size, DGF definition, mean or median NGAL levels of DGF and Non-DGF groups, the time of obtaining specimen, cold ischemia time (CIT), expanded criteria donor (ECD) or donor after cardiac death (DCD) ratio, donor age and donor terminal Scr level. (2) Predictive values, including sensitivity, specificity, cut-off value and AUC with 95% confidence interval (CI). If multiple AUC values of different sampling time points were provided in the same study, the AUC values of 24 h and 48 h were recorded. In addition, if there were multiple cut-off values at the same sampling time point, the one showing the highest Youden index (sensitivity + specificity-1) was extracted. We evaluated the quality of the included articles via Quality Assessment of Diagnosis Accuracy Studies-2 (QUADAS-2) tool that comprises 14 questions [6]. “Yes”, “Unclear” and “No” were chosen for each signaling question in the context of original articles.

### Statistical analysis

Based on the heterogeneity of included studies, a random effect model (DerSimonian Laird) or fixed effect model (Mantel-Haenszel) was employed to estimate the pooled sensitivity, specificity and diagnostic odds ratio (DOR) with 95% CI. Moreover, forest plots of sensitivity, specificity, DOR and summary ROC (SROC) with overall AUC value were presented. Cochran’s *Q* test was used to assess heterogeneity across studies and inconsistency index *I*^*2*^ was applied to estimate the degree of heterogeneity. I^2^ values of 25, 50 and 75% were thought to be low, moderate and high heterogeneity, respectively [7]. When substantial heterogeneity was found (I^2^ > 50%), Spearman correlation coefficient test was performed to detect the presence of threshold effect; Sensitivity analysis was conducted to explore the source of non-threshold effect heterogeneity. Publication bias was statistically assessed by Deeks’ linear regression test. In addition, we compared the risk factors of DGF (including DCD or ECD ratio, donor age, donor Scr and CIT) between uNGAL and bNGAL studies to figure out whether they were significantly different between these two groups of studies. The overall levels of these risk factors in each study were calculated using available mean or median values and patient numbers from DGF and Non-DGF groups. Student’s t-test or Mann-Whitney U-test were utilized to compare continuous variables with normal distribution and skewed distribution, respectively. All statistical analyses were performed with Stata version 12.0 (Stata Corporation, College Station, Texas) and SPSS version 23.0 (SPSS Inc., Chicago, IL, USA). A two tailed *P*-value less than 0.05 was considered statistically significant.

## Results

### Search results and study selection

**Fig. 1:**
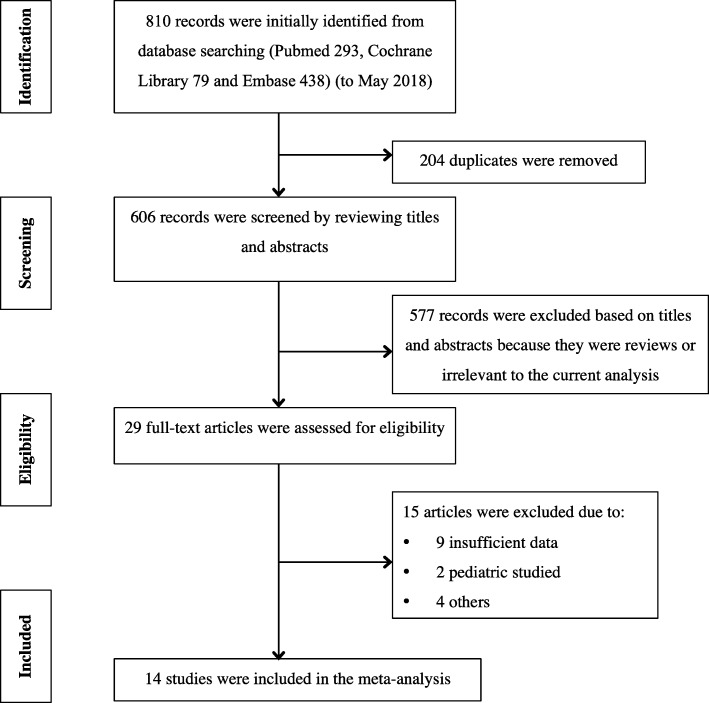
Flow chart of study selection

Our initial search yielded 810 records. After removing all duplicates, 606 records remained for further screening. By looking through titles and abstracts, the remaining 29 articles were evaluated in full-text manner. Eventually, 14 eligible articles [8–21] were accepted in the meta-analysis according to pre-established inclusion criteria. No additional articles were found by sifting for titles and abstracts of the references in eligible articles and most related reviews. Detailed flow diagram of study selection is shown in Fig. 1.

### Study characteristics

Characteristics of individual study were summarized in Table 1. 14 eligible articles from 10 different countries were published between 2006 and 2016. All studies were prospective cohort studies except for 2 studies that were designed in retrospective manner [12, 13]. Eight studies including 10 datasets (seven 24 h uNGAL and three 48 h uNGAL) evaluated uNGAL and six studies evaluated bNGAL (three serum and three plasma). The studies encompassed a total of 1036 kidney transplant recipients [median sample size, 55(38–91)], among whom 333 recipients [median sample size, 15 (13–22)] developed DGF. Males represented 61.4% of the study population. 9 studies defined DGF as the requirement for dialysis within 7 days after transplantation, 4 studies [13, 17–19] combined the dialysis-based criterion and the failure of Scr to decrease or the graft histopathology criterion to define DGF and pezeshgi’s study [20] did not describe the detailed definition of DGF. No significant differences were observed between 8 uNGAL studies and 6 bNGAL studies in terms of DCD or ECD ratio, donor age, donor Scr and CIT (*P* > 0.05).

**Table 1:** Characteristics of 14 included studies

Study (y)	Design	Country	Sample type	Sample size, N	Male (%)	DGF, N (%)	Donor type (DCD/ECD,%)	Donor age, year (DGF/Non-DGF)	Donor Scr, umol/l (DGF/Non-DGF)	CIT, min (DGF/Non-DGF)	NGAL, mg/ml (DGF/Non-DGF)	DGF definitions
Urine NGAL												
Parikh (2006) [8]	PC	USA	Urine	30	46.7	10 (33.3)	56.6	NR	NR	1176 ± 612/990 ± 336	3360 (17–5850)/ 756 (12–2500)^**#**^	Dialysis within 1 week
Hall (2010) [9]	PC	USA	Urine	91	63	34 (37.4)	100	NR	NR	NR	1035 (95–3134)/NR	Dialysis within 1 week
Hollmen (2011) [11]	PC	Finland	Urine	176	62.5	66 (37.5)	100	56 (9–75)/ 49 (9–75)*	64 ± 17/63 ± 21	1374 ± 216/1278 ± 222*	931 ± 715.1/NR	Dialysis within 1 week
Kanter (2013) [21]	PC	Spain	Urine	38	52.6	15 (39.5)	37.5	54 ± 12/43 ± 14	NR	1038 ± 342/1044 ± 228	275 (152–634)/92 (51–444)	Dialysis within 1 week
Fonseca (2013) [14]	PC	Spain	Urine	40	65.0	18 (45.0)	7.5	51 ± 13/51 ± 10	75 ± 19/69 ± 14	912 ± 468/576 ± 438	834 (510–2632)/80 (29–138)	Dialysis within 1 week
Cui (2015) [17]	PC	China	Urine	123	68.3	21 (17.1)	100	NR	NR	720 (600–840) /600 (540–780)	657 (299–1624)/38 (11–120)	Dialysis within 1 week or Scr decreased by < 10% per day
Lacquaniti (2016) [18]	PC	Italy	Urine	29	58.6	22 (75.9)	100	64 ± 8/25 ± 13*	115 ± 61/71 ± 18*	966 ± 246/456 ± 90*	136 ± 93/47 ± 40	Dialysis within 1 week or Scr decreased by < 10% per day
Nieto-Rios (2016) [19]	PC	Clombia	Urine	79	55.7	13 (16.5)	100	27 (24–42)/26 (19–41)	71 (60–85)/71 (53–97)	1092 ± 402/852 ± 329*	NR/NR	Dialysis within 1 week or Scr decreased by < 10% per day
Summary	–	–	–	606	59.1	199 (32.8)	82.4	53.6/41.2	75.9/66.5	1121.2/907.7	–	–
Blood NGAL												
Bataille (2011) [10]	PC	France	Plasma	41	63.4	15 (36.6)	95.1	43 (42–51)/55 (39–63)	126 (86–150)/80 (50–90)	735 (575–797)/889 (662–1020)	571 (467–634)/242 (158–199)	Dialysis within 1 week
Lee (2012) [13]	RC	Korea	Serum	59	59.3	14 (23.7)	52.5	45 ± 11/41 ± 11	NR	337 ± 132/311 ± 107	490 (238–723)/184 (145–233)	Dialysis within 1 week or pathologic findings
Kusaka (2012) [12]	RC	Japan	Serum	67	71.6	13 (19.4)	41.8	47 (15–69)/54	NR	556 (162–972)/310	NR/757 ± 58	Dialysis within 1 week
Hollmen (2014) [15]	PC	Finland	Serum	176	62.5	66 (37.5)	100	56 ± 12/49 ± 15*	64 ± 17/63 ± 21	1374 ± 216/1278 ± 222*	588 ± 190/355 ± 166	Dialysis within 1 week
Cantaluppi (2015) [16]	PC	Italy	Plasma	50	42	14 (28.0)	100	65 ± 9/68 ± 7	67 ± 20/70 ± 34	985 ± 237/1032 ± 177	663 ± 97/380 ± 140	Dialysis within 1 week
Pezeshgi (2016) [20]	PC	Iran	Plasma	37	59.5	12 (32.4)	NR	NR	NR	NR	437 ± 164/252 ± 58	NR
Summary	–	–	–	430	60.9	134 (31.2)	75.9	53.2/51.8	74.2/67.0	1044.6/854.5	–	–
*P* value	**–**	–	–	0.846	0.885	0.330	0.892	0.339	0.936	0.423	–	–

*PC* Prospective cohort, *RC* Retrospective cohort, *NR* not reported

*P < 0.05 between DGF and non-DGF groups; ^**#**^ ng/mg; *P* value for the comparison between uNGAL studies and bNGAL studies

### Quality assessment and publication bias

The quality of studies based on QUADAS-2 tool was summarized in Additional file 1: Figure S1 Deeks’ Funnel plot indicated no significant publication biases were existed among the included studies of both uNGAL and bNGAL (Additional file 2: Figure S2).

### Data synthesis and heterogeneity analysis

**Fig. 2:**
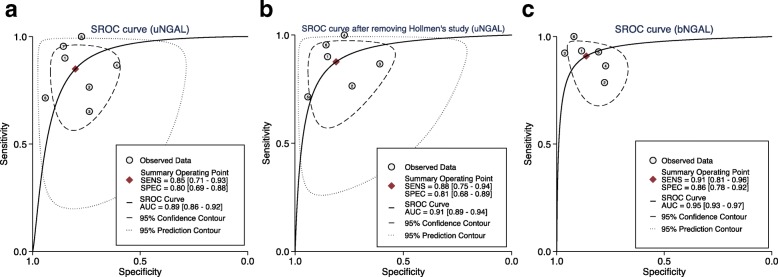
Hierarchical summary receiver operating characteristic (SROC) plots of uNGAL (**a**), uNGAL after removing Hollmen’s study (**b**) and bNGAL (**c**) level to predict DGF in kidney transplant recipients. The curves are represented by the straight lines; Each of the analyzed studies is represented by a circle; the point estimate to which summary sensitivity (SENS) and specificity (SPEC) correspond is represented by the diamond shape, and the respective 95% CI, by the dashed lines, whereas the 95% confidence area in which a new study will be located is represented by the dotted lines

**Fig. 3:**
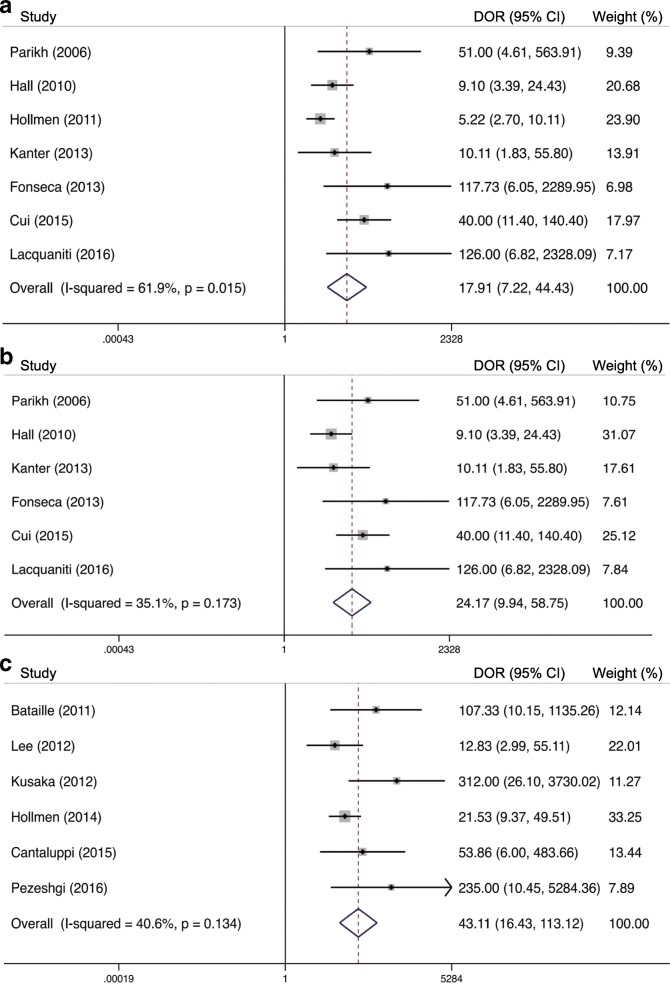
Forest plots of the pooled diagnostic odds ratio (DOR) of uNGAL (**a**), uNGAL after removing Hollmen’s study (**b**) and bNGAL (**c**) in predicting DGF in kidney transplant recipients. The black squares in the gray squares and the horizontal lines represent the point estimate and 95% CI, respectively. The dotted line represents the pooled estimate, and the hollow diamonds represent the 95% CI of the pooled estimate

AUC value, cut-off value, sensitivity and specificity of individual dataset were summarized in Table 2**.** True-positive (TP), false-positive (FP), true-negative (TN) and false-negative (FN) were calculated based on the provided sensitivity, specificity and patient numbers in each study. To compare the predictive performances of uNGAL and bNGAL for DGF, we controlled the sampling time at 24 h. Random effect models were utilized to pool the specificities of 24 h uNGAL (7 datasets) and 24 h bNGAL (6 datasets), sensitivity and DOR for 24 h uNGAL because the obvious heterogeneities were observed (I^2^ > 50%, *P* < 0.05). Fix effect models were used to summarize the sensitivity and DOR for 24 h bNGAL (I^2^ < 50%, *P* > 0.05). The composite AUC for uNGAL was 0.89 (95% CI, 0.86–0.92) and the overall DOR for uNGAL was 17.91 (95% CI, 7.22–44.43) with a sensitivity of 0.85 (95% CI, 0.71–0.93) and a specificity of 0.80 (95% CI, 0.69–0.88). The composite AUC for bNGAL was 0.95 (95% CI, 0.93–0.97) and the overall DOR for bNGAL was 43.11 (95% CI, 16.43–113.12) with a sensitivity of 0.91 (95% CI, 0.81–0.96) and a specificity of 0.86 (95% CI, 0.78–0.92). Pooled SROC curves and DOR of 24 h uNGAL and 24 h bNGAL were plotted in Fig. 2 ***(a, c)*** and Fig. 3 (a, c), respectively. Pooled sensitivities and specificities for 24 h uNGAL and 24 h bNGAL were both presented in Additional file 3: Figure S3*.*

**Table 2:** Predictive values of urine and serum/plasma NGAL for DGF in individual studies

Study (y)	Sampling Time (h)	AUC (95% CI)	Sensitivity (%)	Specificity (%)	Cut-off (ng/ml)	TP	FP	FN	TN	Sample size
Urine NGAL										
Parikh (2006) [8]	24	0.90 (0.71–1.00)	90.0	83.0	1000^**#**^	9	3	1	17	30
Hall (2010) [9]	24	0.82 (0.72–0.92)	65.0	94.0	800	26	15	8	42	91
Hollmen (2011) [11]	24	0.74 (0.64–0.83)	65.0	74.0	560	43	29	23	81	176
Kanter (2013) [21]	24	0.71 (0.51–0.91)	85.7	61.5	128	13	9	2	14	38
Fonseca (2013) [14]	24	0.88 (0.77–1.00)	100.0	76.0	286	18	5	0	17	40
	48	0.96 (0.90–1.00)	93.0	90.0	277	17	2	1	20	40
Cui (2015) [17]	24	0.834 (0.677–0.992)	70.0	93.7	688.3	15	6	6	96	123
	48	0.897 (0.764–0.969)	80.0	96.9	295.2	17	3	4	99	123
Lacquaniti (2016) [18]	24	0.97 (0.90–0.99)	95.8	91.9	105	21	1	1	6	29
Nieto-Rios (2016) [19]	48	0.80 (NR)	75.0	70.0	120	10	20	3	46	79
Serum or plasma NGAL										
Bataille (2011) [10]	24 h	0.97 (0.93–1.00)	93.3	88.5	400	14	3	1	23	41
Lee (2012) [13]	24 h	0.86 (0.75–0.98)	78.6	77.8	233.3	11	10	3	35	59
Kusaka (2012) [12]	24 h	0.99 (NR)	91.0	97.0	500	12	2	1	52	67
Hollmen (2014) [15]	24 h	0.85 (0.79–0.91)	87.0	77.0	423	57	25	9	85	176
Cantaluppi (2015) [16]	24 h	0.94 (NR)	90.9	80.6	532	13	7	1	29	50
Pezeshgi (2016) [20]	24 h	0.97 (NR)	100	92	317	12	2	0	23	37

*TP* true-positive, *FP* false-positive, *FN* false-negative, *TN* true-negative, *NR* not reported; ^#^ng/mg

**Fig. 4:**
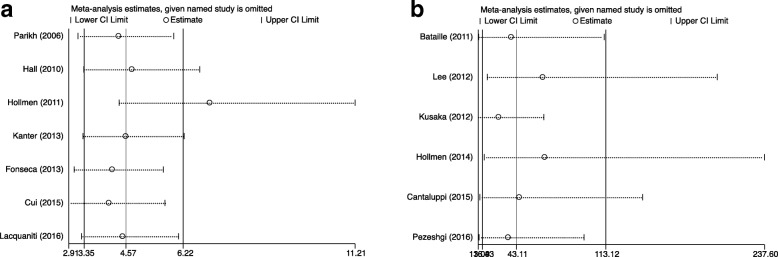
Sensitivity analysis after each study was excluded by turns. (**a**) for uNGAL and (**b**) for bNGAL

To explore possible reasons for heterogeneity, Spearman correlation coefficient tests and Sensitivity analyses were performed. No significant threshold effect was observed among bNGAL studies (*r* = 1.00, *P* = 1.00). While the coefficient of 7 datasets analyzing 24 h uNGAL (*r* = − 0.10, *P* = 0.01) indicated the presence of threshold effect. Therefore, hierarchical SROC modeling was plotted to pool the sensitivity and specificity [22]. Sensitivity analyses were conducted by omitting study one by one to investigate the source of non-threshold effect heterogeneity across studies. As demonstrated in Fig. 4a, the estimate value of uNGAL was beyond the limitation of upper CI when removing Hollmen’s (2011) study, suggesting that Hollmen’s (2011) study was the main source of the heterogeneity. Then, we performed the pooled analysis without including Hollmen’s (2011) study. The results revealed that the composite AUC for uNGAL was 0.91 (95% CI, 0.89–0.94); the overall DOR was 43.11 (95% CI, 16.43–113.12) with I^2^ value dropped from 61.9 to 35.1%; the sensitivity was 0.88 (95% CI, 0.75–0.94) and the specificity was 0.81 (95% CI, 0.68–0.89) (Figs. 2b 3b, 4b showed an overlapping CI, enhancing the stability of our data synthesis for bNGAL.

## Discussion

Previously, several meta-analysis and systematic reviews have summarized the significance of uNGAL and bNGAL in predicting acute kidney injury (AKI) in different clinical settings, such as in cardiac surgery [23], contrast-induced AKI after cardiac catheterization [24] and found they were promising biomarkers for early detection of AKI. However, their predictive ability for DGF, a form of AKI post-transplantation, varied among different studies. So we quantitatively investigated the predictive values of uNGAL and bNGAL for the early (24 h) identification of DGF in kidney transplant recipients in this study. The results suggested that both uNGAL and bNGAL were valuable renal biomarkers for predicting DGF with high AUC values of 0.91 and 0.95 respectively. In addition, bNGAL appeared to be a better biomarker than uNGAL in predicting DGF with consideration of AUC values, sensitivities and specificities.

NGAL, also known as lipcalin-2 (LCN2), is secreted by various tissues such as kidney tubules, liver, lung and gastrointestinal tract at a low level in healthy controls [25]. It has been reported to involve in several pathways such as apoptosis, bacteriostasis, renal tubule epithelial cell proliferation and regeneration [26]. NGAL has several forms including monomeric froms, dimers and trimers. The majority of NGAL was in monomeric form (with a molecular weight of 25 kDa) that was mainly produced by injured kidney tubule epithelium [27]. When there is proximal tubular injury, NGAL levels would increase rapidly. So NGAL has emerged as a promising predictor of AKI in recent years and been regarded as the “troponin of kidney” [28].

In kidney transplantation, uNGAL and bNGAL have been extensively studied in the prediction and diagnosis of short- and long-term renal functions [29]. The sources of NGAL are different in urine and blood. Most uNGAL was derived from distal nephron synthesis rather than filtered from blood, while bNGAL not only came from the damaged kidney, but also the systematic pool [30]. So, theoretically, uNGAL was expected to be more representative of kidney injury than bNGAL. However, this hypothesis was not borne out in our analysis of DGF. The results of the current meta-analysis demonstrated that bNGAL had a pooled DOR of 43.11 that was almost twice as higher as uNGAL (DOR, 24.17). Moreover, bNGAL showed an obviously higher sensitivity and a slightly higher specificity than those of uNGAL, which supported the superiority of bNGAL over uNGAL in predicting DGF within 24 h in kidney transplant recipients. This was consistent with Buemi’s study [31], which investigated the predictive values of uNGAL and plasma NGAL (pNGAL) for DGF in 97 recipients and found that pNGAL was superior to uNGAL in terms of AUC values. In addition, two studies [11, 15] from Hollmen’s group separately explored the predictive potential of uNGAL and serum NGAL for DGF in the same kidney transplant recipient cohort and found higher AUC value of sNGAL (AUC, 0.85) compared to uNGAL (AUC, 0.74). One of the possible reasons was that many factors (such as urine concentration and glomerular filtration rate) might affect the levels of uNGAL, thus weakening the predictive ability of uNGAL. These findings were interesting and had great significance. Because in addition to its greater predictive performance, bNGAL would be more feasible for kidney transplant recipients when they suffered from a severe condition of oliguria and even anuria post-operatively. However, due to the inconsistent cut-off values and unavailability of original data from included studies, we were unable to determine the optimal predictive cut-off value for bNGAL to be implement in clinical practice.

Determining when to detect NGAL is important for clinicians to identify DGF effectively in kidney transplant recipients. It was reported that NGAL began to rise 2 h after the injury, peak at 8-12 h and return after 24-48 h [9]. So we recorded the prediction data when the sampling time was within 24 h and within 48 h, trying to explore the impact of different sampling time on predictive performance of NGAL. Qualitative analysis of Fonseca’s (2013) [14] and Cui’s (2015) [17] studies (simultaneously analyzed the 24 h and 48 h uNGAL) demonstrated that 48 h uNGAL showed larger AUCs and better sensitivity and specificity than those of 24 h uNGAL, while Nieto-Rios’s (2016, 19] (only detected 48 h uNGAL) presented relatively lower AUC of 0.80. It was inappropriate to draw any conclusion on this issue due to the limited data and the different study designs of these studies. Large prospective kidney transplant cohorts with different sampling time points are required to elucidate the features of early and late NGAL in predicting the risk of DGF. However, from a practical point of view, early results obtained a few hours after transplantation have more potential to alert clinicians to evaluate the risk of DGF and anticipate therapeutic intervention. So we believe that it is more valuable to detect NGAL within first 24 h or to monitor NGAL dynamically.

Limitations in this meta-analysis should be mentioned here. Firstly, the inconsistence of DGF definition may alter the predictive performance of uNGAL and bNGAL for DGF in individual studies, thereby may bring some deviations. Secondly, we only included the published literatures whose predictive variables (TP, TN, FP and FN) were available or can be calculated. So the predictive performance of uNGAL and bNGAL may be overestimated due to the publication and selection bias. Thirdly, different cut-off values were used in the included studies. These discrepancies may be attributed to the differences in experimental designs including detection methods, sample size and patient baseline characteristics, which may affect the sensitivity and specificity in individual study. To find an optimal cut-off value that can be implemented in clinical settings, further investigation in large prospective cohorts are required. Importantly, it might be necessary for each research center to pre-define specific normal ranges for urine and blood NGAL in normal kidney transplant recipients before its clinical application.

## Conclusions

In conclusion, the results of the present meta-analysis demonstrated that both urine NGAL and blood NGAL appeared to be valuable biomarkers of DGF in adult kidney transplant recipients. In addition, the blood NGAL was superior to urinary NGAL in early prediction of DGF. However, further large-scale prospective cohort studies are needed to determine the optimal cut-off value for NGAL that is suitable for clinical use.

## Additional files

Additional file 1: **Figure S1.** Quality assessment of the 14 included studies using QUADAS-2 tool. The assessment of risk of bias and applicability concerns for individual study (PDF 98 kb)

Additional file 2: **Figure S2.** Deeks’ funnel plots for the evaluation of potential publication bias in prediction of uNGAL (**a**) and bNGAL (**b**) for DGF (PDF 876 kb)

Additional file 3: **Figure S3.** Forest plots of the pooled sensitivities and specificities of uNGAL (**a**) and bNGAL (**b**) level in predicting DGF in kidney transplant recipients. The black squares in the gray squares and the horizontal lines represent the point estimate and 95% CI, respectively. The dotted lines represent the pooled estimate, and the hollow diamonds represent the 95% CI of the pooled estimate (PDF 5145 kb)

## Data Availability

All data analyzed during this study are included in this article and in the supplementary files.

## Abbreviations

AKI: Acute kidney injury
AUC: Area under the receiver operating characteristic curve
b: blood
CI: Confidence interval
CIT: Cold ischemia time
DCD: Donor after cardiac death
DGF: Delayed graft function
DOR: Diagnostic odds ratios
ECD: Expanded criteria donor
FN: False-negative
FP: False-positive
LCN2: Lipcalin-2
NGAL: Neutrophil gelatinase associated lipocalin
p: plasma
QUADAS-2: Quality Assessment of Diagnosis Accuracy Studies-2
Scr: Serum creatinine
SROC: Summary receiver operating characteristic curves
TN: True-negative
TP: True-positive
u: urinary
