# Supplementary material for: Comparison of urine and blood NGAL for early prediction of delayed graft function in adult kidney transplant recipients: a meta-analysis of observational studies
Source: BMC Nephrol. 2019 Aug 2;20:291. doi: 10.1186/s12882-019-1491-y (PMC6679493; doi:10.1186/s12882-019-1491-y)
Supplement: Supplementary file 1 — Figure S1. Quality assessment of the 14 included studies using QUADAS-2 tool. The assessment of risk of bias and applicability concerns for individual study (PDF 98 kb) [file 12882_2019_1491_MOESM1_ESM.pdf]

	Risk of Bias				Applicability Concerns		
	Patient Selection	Index Test	Reference Standard	Flow and Timing	Patient Selection	Index Test	Reference Standard
Bataille (2011)	+	+	?	–	+	+	?
Cantaluppi (2015)	?	+	+	+	?	+	+
Cui (2015)	?	+	?	–	?	+	?
Fonseca (2013)	+	+	+	+	+	+	+
Hall (2010)	+	+	+	+	+	+	+
Hollmen (2011)	+	+	+	+	+	+	+
Hollmen (2014)	+	+	+	+	+	+	+
Kanter (2013)	?	+	+	+	+	+	+
Kusaka (2012)	–	–	+	+	–	+	+
Lacquaniti (2016)	+	+	?	–	+	+	?
Lee (2012)	–	+	?	?	?	+	+
Nieto-Ríos (2016)	+	+	?	+	+	+	?
Parikh (2006)	+	+	+	+	+	+	+
Pezeshgni (2016)	?	+	?	?	+	+	?

– **High** ? **Unclear** + **Low**
