# Supplementary figures and images for: Comparison of urine and blood NGAL for early prediction of delayed graft function in adult kidney transplant recipients: a meta-analysis of observational studies
Source: BMC Nephrol. 2019 Aug 2;20:291. doi: 10.1186/s12882-019-1491-y (PMC6679493; doi:10.1186/s12882-019-1491-y)

**a**

Deeks' Funnel Plot (uNGAL)  
P value = 0.12

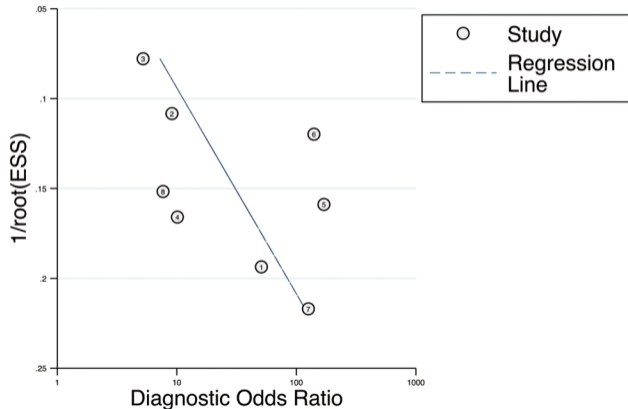**b**

Deeks' Funnel Plot (bNGAL)  
P value = 0.16

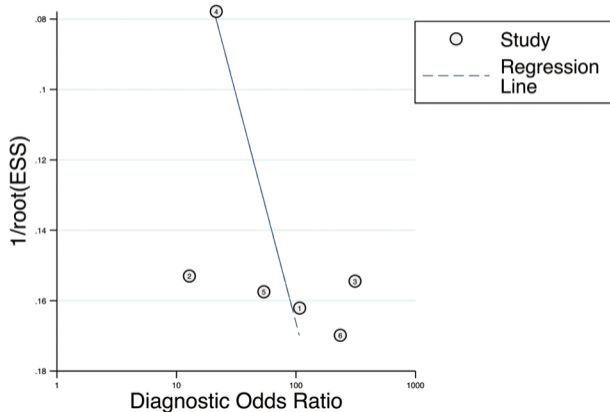

Supplement: Supplementary file 2 — Figure S2. Deeks’ funnel plots for the evaluation of potential publication bias in prediction of uNGAL (a) and bNGAL (b) for DGF (PDF 876 kb) [file 12882_2019_1491_MOESM2_ESM.pdf]

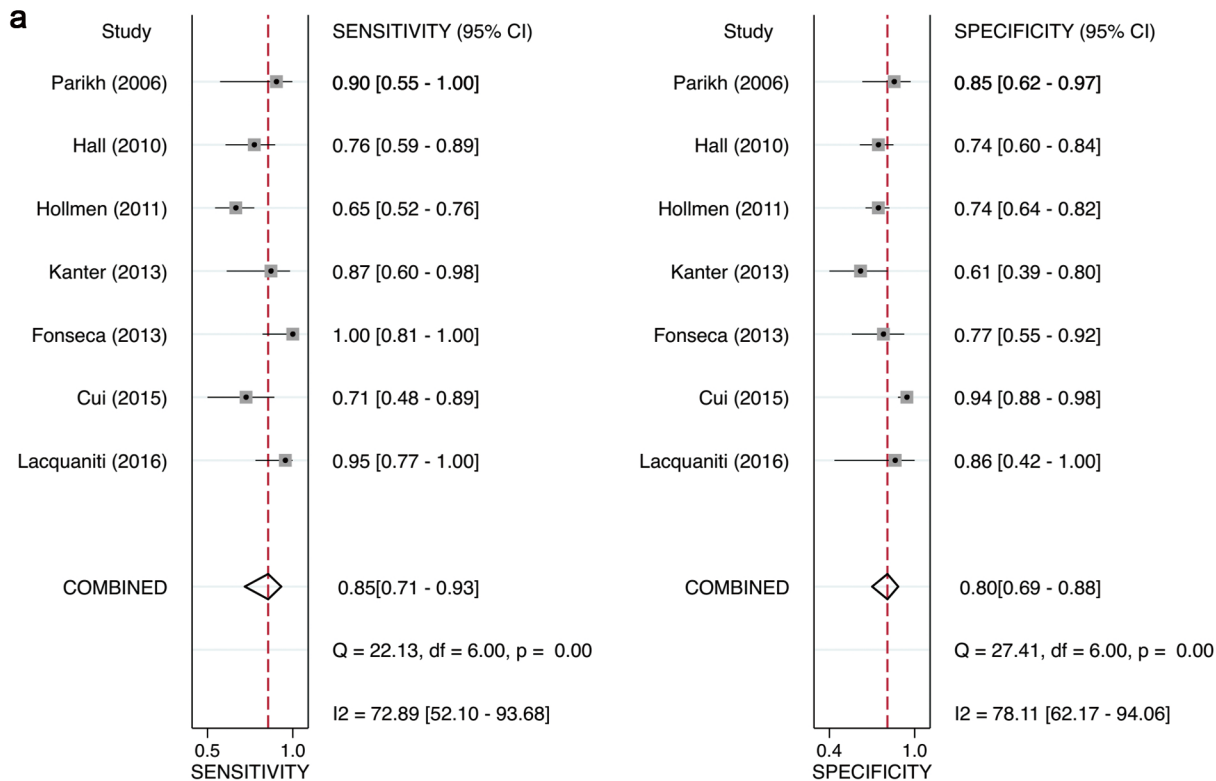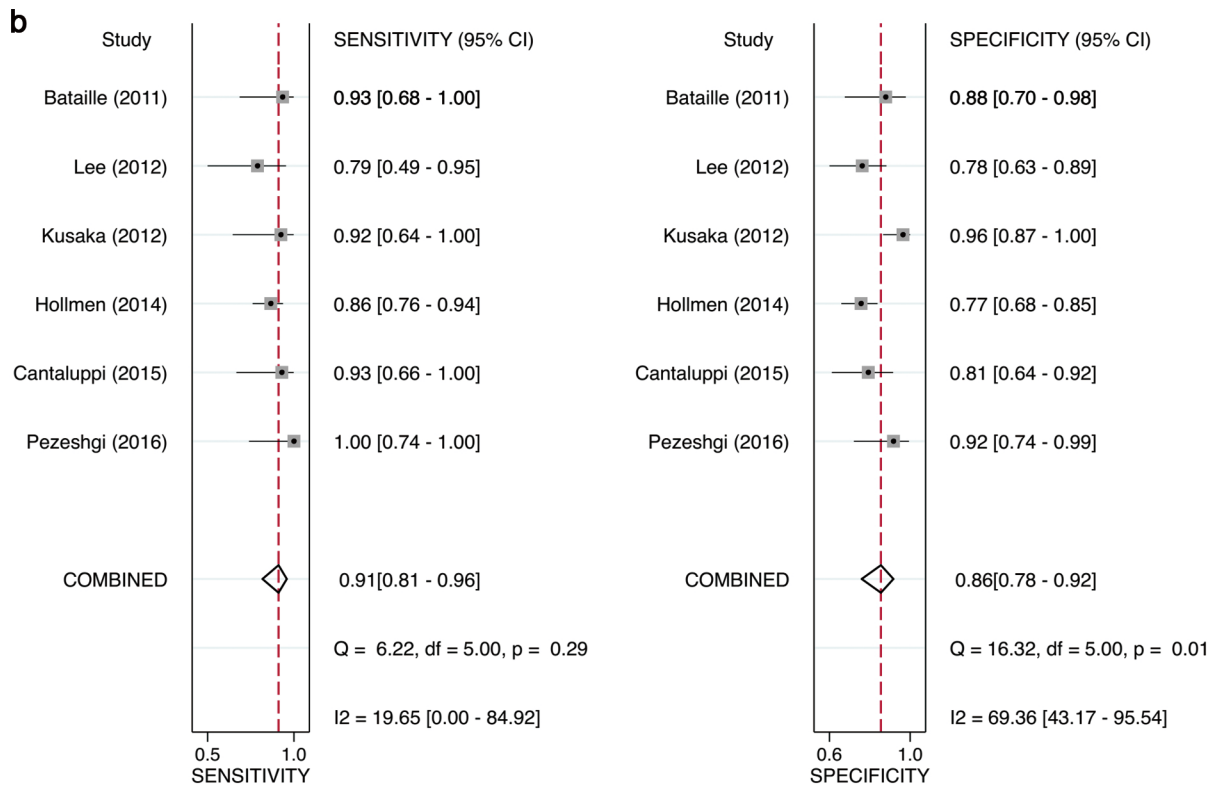

Supplement: Supplementary file 3 — Figure S3. Forest plots of the pooled sensitivities and specificities of uNGAL (a) and bNGAL (b) level in predicting DGF in kidney transplant recipients. The black squares in the gray squares and the horizontal lines represent the point estimate and 95% CI, respectively. The dotted lines represent the pooled estimate, and the hollow diamonds represent the 95% CI of the pooled estimate (PDF 5145 kb) [file 12882_2019_1491_MOESM3_ESM.pdf]
